# The direct and indirect effects of the pneumococcal conjugated vaccine on carriage rates in children aged younger than 5 years in Latin America and the Caribbean: a systematic review

**DOI:** 10.31744/einstein_journal/2020RW4890

**Published:** 2019-11-08

**Authors:** Stefany Martins Silva, Izabella Caroline Gebrim Rodrigues, Rodrigo da Silva Santos, Yves Mauro Fernandes Ternes

**Affiliations:** 1 Universidade Federal de Goiás, Goiânia, GO, Brazil.

**Keywords:** Vaccines, Streptococcus pneumoniae, Carrier state, Child, Latin America

## Abstract

**Objective:**

To demonstrate the impact of pneumococcal conjugate vaccine in *Streptococcus pneumoniae* carriage status in children younger than 5 years in Latin America and the Caribbean.

**Methods:**

A systematic literature review was carried out on the direct and indirect effects of pneumococcal vaccine in the carriage status, after implementation in childhood immunization programs. Studies carried out in children younger than 5 years were selected from the PubMed^®^ and Virtual Health Library databases, and data collected after implementation of pneumococcal vaccine in Latin America and the Caribbean, between 2008 and 2018.

**Results:**

From 1,396 articles identified, 738 were selected based on titles and abstracts. After duplicate removal, 31 studies were eligible for full-text reading, resulting in 6 publications for analysis. All selected publications were observational studies and indicated a decrease in the carriage and vaccine types, and an increase in the circulation of non-vaccine serotypes, such as 6A, 19A, 35B, 21 and 38. We did not identify changes in the antimicrobial resistance after vaccine implementation.

**Conclusion:**

A decrease in the carriage status of vaccine types and non-vaccine types was detected. The continuous monitoring of pneumococcal vaccine effect is fundamental to demonstrate the impact of the carriage status and, consequently, of invasive pneumococcal disease, allowing better targeting approaches in countries that included pneumococcal vaccine in their immunization programs. Our study protocol was registered in PROSPERO (www.crd.york.ac.uk/prospero) under number CRD42018096719.

## INTRODUCTION

The World Health Organization (WHO) characterizes respiratory conditions, notably pneumococcal diseases, as the leading cause of morbidity and mortality in childhood.^1 , 2^ The pneumococcal conjugate vaccine (PCV) is a remarkable advance for public health, and it is the primary prevention method against pneumococcal diseases in vulnerable populations, such as children younger than 5 years.^2^

The WHO suggests developing countries should be prioritized in the effort to reduce the mortality rate of children aged under 5 years, since this population is often immunocompromised and malnourished. In addition, these individuals come from low-income families, present several comorbidities, and generally live in precarious conditions. These factors indicate that adding PCV to the childhood immunization programs of developing countries would remarkably benefit this population.^3^ In 2010, Brazil was one of the first Latin American countries to include a PCV, the 10-valent pneumococcal vaccine (PCV10), on the national immunization program. Pneumococcal conjugate vaccine 10 is composed of the top ten serotypes of *Streptococcus pneumoniae* (pneumococcus) more frequently associated with pneumococcal diseases in the country.^4^ Other Latin American countries, such as Chile, established the use of the 13-valent pneumococcal vaccine (PCV13) to their immunization program, in 2010.^3^

*S. pneumoniae* is a significant respiratory pathogen capable of colonizing the respiratory tract mucosa and the nasopharyngeal region. This carriage status can last from weeks to months. The agent presents a polysaccharide capsule with more than 90 capsular serotypes,^5 , 6^ and can be transmitted through direct contact with secretions or aerosol dispersion by affected individuals.^7^

Therefore, after implementation of PCV in the national immunization programs, it is expected that pneumococcus carriage status will diminish, which may reduce pneumococcal infection burden amongst children. The effect in the carriage status may be a scenario of serotype substitution.^8^ By taking these hypotheses together, the objective of this study was to investigate the direct and indirect effects of the pneumococcus carriage status in children younger than 5 years. Determining pneumococcus nasopharyngeal colonization in this population may generate insights for surveillance and strategical policies in countries that implemented PCV in their national immunization programs.

## OBJECTIVE

To demonstrate the impact of pneumococcal conjugate vaccine in *Streptococcus pneumoniae* carriage status of children younger than 5 years in Latin America and the Caribbean.

## METHODS

We performed a systematic review of the literature with studies conducted in Latin America and the Caribbean (LA&C), published between January 2008 and February 2018. We selected scientific publications related to the impact (direct and indirect effects) of the pneumococcal vaccine in the carriage status of pneumococcus in children after the implementation of PCV in the childhood immunization programs. The target age groups for PCV were assessed as a direct effect; the non-target age groups for PCV were considered as indirect effect of the vaccine.

Searches were performed at National Center for Biotechnology Information, National Library of Medicine (NCBI/PubMed^®^) and Virtual Health Library (VHL) databases. We did not employ language restrictions to the studies. Publications not fully available at the platforms were not evaluated.

### Inclusion and exclusion criteria

We investigated studies conducted in LA&C countries and published between 2008 and 2018, provided the data collection had been performed after PCV implementation. For eligibility of our literature review, we exclusively evaluated studies conducted with children younger than 5 years, and presenting results of carriage status. To this end, only observational studies were taken into account.

We excluded from our analyses review papers, randomized and experimental studies, studies performed in countries outside LA&C, publications including children over 5 years of age, studies published before 2008, and that contained outcome for invasive diseases.

### Selection process

To select the studies from the publication databases, we used the following keywords: (pneumococcal vaccine AND children AND carriage) OR (pneumococcal vaccine AND children AND colonizing) OR ( *Streptococcus pneumoniae* vaccine AND children AND carriage) OR ( *Streptococcus pneumoniae* vaccine and children AND colonizing). The selected studies were analyzed by two independent reviewers. We prepared two forms that were used to stratify the selected manuscripts based on The Professional Society for Health Economics and Outcomes Research (ISPOR) reports. In the Level 1 Data Extraction Form, studies were screened based on titles and abstracts, using the exclusion criteria above mentioned. The selected studies were then subjected to analysis through Level 2 Data Extraction Form, which was based on the full-text screening. During eligibility analysis, a third reviewer analyzed the studies that were discordant between the two first reviewers. Forms 1 and 2 are available in the supplementary material.

## RESULTS

**Figure 1:**
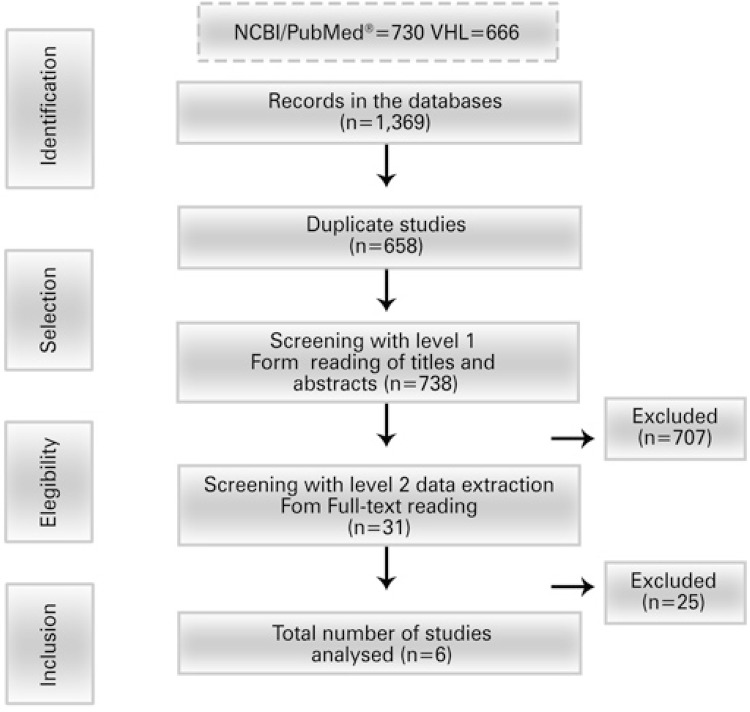
Study selection flow

The flow for identifying and selecting studies is shown in figure 1 . We identified 1,396 studies and excluded 658 duplicates. After triage with the Level 1 Data Extraction Form, from 738 studies investigated, 31 met all required criteria and were then subjected to the Level 2 Data Extraction Form. From the 31 reports, 25 were characterized as potentially eligible, unclear or excluded, resulting in 6 studies for analysis. Out of six studies selected, three were carried out in Brazil, one in Colombia, one in Venezuela, and one in Peru. The majority of studies (four studies) compared the pre and post-vaccination periods, with different PCV. Therefore, the individual effect of each country/PCV should be taken into account as a means of suggesting the increase in valence of the vaccine, without comparing the effect among the countries.

The features of each study are shown in table 1 . The Brazilian studies investigated the effects after the introduction of the PCV10 in the National Childhood Immunization Program, in 2010.^9^ The study from Venezuela was carried out with nasopharyngeal samples of 84 children younger than 5 years, who were vaccinated with PCV7.^10^ Pneumococcal conjugate vaccine 7, which was distributed throughout the Latin American territory, was not included in the immunization program of Venezuela. However, its benefits, effects, and serotypes found in circulation were investigated. In 2014, PCV13 was implemented in the immunization program and was offered to the child population of Venezuela.^11 , 12^ The studies from Colombia and Peru characterized the effects of PCV7 after the introduction of this vaccine in the immunization program of both countries, in 2009.^13 , 14^

**Table 1:** Direct and indirect effects of the selected studies

Reference	Country (city)	Population (n) ^post-vaccine^	Vaccine (years of investigation)	Effect	Resistance to antibiotics
Bello González et al.^(10)^	Venezuela (Bolívar)	<5 years (n=84)	PCV 7^*^	High prevalence of the non-vaccine serotype 33F serotype (21.5%) in the carriage rates	No resistance to penicillin was detected
			Post-vaccine (2008)		
Parra et al^.(13)^	Colombia (Bogotá)	12-18 months (n=197)	PCV7	Reduction of vaccine serotype^†^ PCV7 (23.6% non-immunized to 7.6% in immunized children)	Resistance verified for invasive disease
			Pre- and post-vaccine (2005-2009/2011)		
				Increase in non-vaccine serotype^‡^ (24% non-immunized to 34% in immunized children)	
Hanke et al.^(14)^	Peru (San Marcos)	Up to 2 years (n=125)	PCV7	Reduction of vaccine serotype^†^ PCV7 (48% → 28.8%) in immunized children	Resistance to antibiotics did not change after vaccine implementation
			Pre- and post-vaccine (2009/2011)		
				Increase in non-vaccine serotype^‡^ (52% → 71.2%) in immunized children	
Andrade et al.^(16)^	Brazil (Goiânia)	7-11 months/15 to 18 month (n=1,287)	PCV10	Reduction of carriage status	Resistance to antibiotics was not verified
			Post-vaccine (2010)	Reduction of vaccine serotype^†^ of PCV10	
Brandileone et al.^(17)^	Brazil (São Paulo)	12-23 months (n=400)	PCV10	Reduction of carriage status (>90%)	Resistance to antibiotics was not verified
			Pre- and post-vaccine (2010/2013)	Reduction of vaccine serotype^†^ PCV10	
Andrade et al.^(18)^	Brazil (Salvador)	6-23 months (n=53)	PCV10	No change was observed in the carriage status	Resistance to antibiotics was not verified
			Pre- and post-vaccine (2009/2013)		

^*^ After use of PCV7 in the territory, since PCV13 was implemented in 2014 in the immunization program; ^†^ vaccine type; ^‡^ non-vaccine type.

PVC: pneumococcal vaccine.

Children living in isolated regions of Bolivia and Venezuela had a higher prevalence of *S. pneumoniae* when between one and two years of age (82%). The serotype 6B was the most frequent (49%), followed by the serotypes 33F (21.5%), 19F (3.1%) and 23F (1.3%). The serotypes 6B and 23F were present in the PCV7 vaccine, referred to as vaccine serotypes, accounting for 51% of serotypes found in the nasopharyngeal of this population. However, the non-vaccine serotype 33F presented high incidence, and was identified in the majority of communities investigated. This observation is highly relevant since 33F serotype rates are increasing all over the world.^10 , 15^

**Figure 2:**
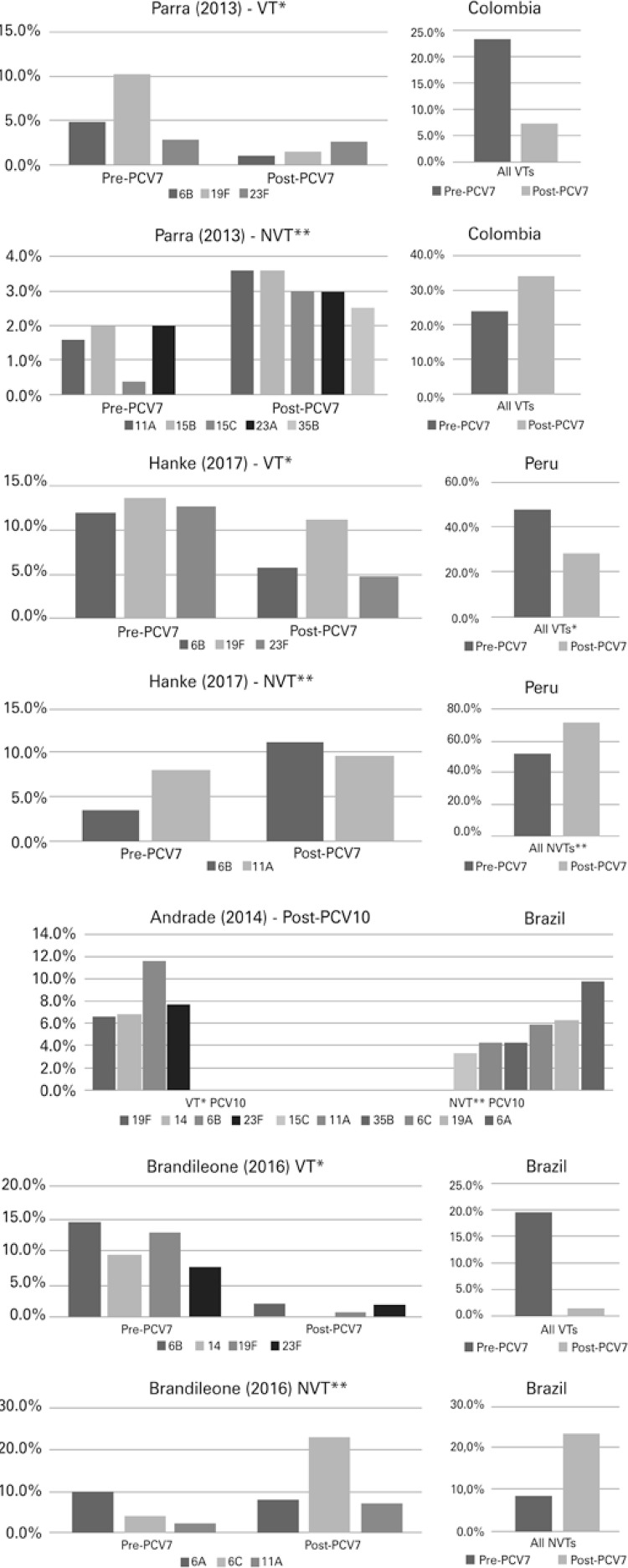
Prevalence of pneumococcal vaccine and nonvaccine types VT: vaccine type; NVT: non-vaccine type.

The associations between the distribution of pneumococcus serotypes and carriage status are shown in figure 2 . The entire set of selected studies reported a significant decrease in the prevalence of vaccine serotypes in the carrier after implementation of the PCV, regardless of the vaccine (PCV7, PCV10 or PCV13). In addition, the substitution effect was evident, since all studies found an increased prevalence of the non-vaccine serotypes.

By investigating the impact of the microbial resistance in the study from Venezuela, none of the strains was resistant to penicillin. Moreover, 13.7% of patients were resistant to macrolides, 3.4% to tetracycline (TET), and 3.4% to trimethoprim-sulfamethoxazole (TMP-STX) and no multi-resistance was found in the evaluated isolates.^10^

## DISCUSSION

To our knowledge, this is the first systematic review of the impact of the PCV on the pneumococcus carriage status in children younger than 5 years, in Latin America and the Caribbean. In our findings, we demonstrated that PCV led to a reduction of the carriage status, which also promoted a lower circulation of the pneumococcus vaccine serotypes in the child population. The decline of the carriage status directly influences the development and mortality as a result of the invasive pneumococcal disease (IPD).^19 - 21^

In general, the studies evidenced a decreased vaccine serotype and increased non-vaccine serotype.^13 , 14 , 16 , 17^ Non-vaccine serotype 33F was exclusively found in Venezuela, with a significant prevalence.^10^ Additionally, Brazil exclusively presented the non-vaccine serotype PCV10 19A serotype, which was increased in the post-vaccination period, similarly to non-vaccine serotype 23A rates in Colombia.^13 , 16^ Likewise, the United States had a substitution of vaccine serotype by non-vaccine serotype, with an increase in the serotypes 6C, 1Z5A, 15B, 15C, 23A and 35B.^22 , 23^ In Europe, studies from Norway and Germany reported that 15A and 23B non-vaccine serotypes were increasing in frequency.^24 , 25^ In France, the non-vaccine serotypes 12F, 15A, 24F and 35B were detected.^26^ In Asia, Japan also had an increase of non-vaccine serotypes, such as 19A, 15A, 15B, 15C, 22F and 24F, after PCV7 implementation, with an non-vaccine serotype increase of 8.0% to 48.1%.^27^

Thus, although the success of PCV in reducing the acquisition of pneumococci that colonize the nasopharyngeal and consequently promote an increase in the IPD load, the PCV etiological profile may be altered due to the increase in carriage status by serotypes absent in the vaccines. This profile alteration of PCVs is referred to as replacement.^8^

The use of vaccines on preventing IPD promoted a decrease in the colonization and circulation of bacterial agents, consequently providing a reduced use of antimicrobial drugs. In this manner, it is possible to diminish the prevalence of resistant or multi-resistant bacteria.^28^ By reducing the number of resistant strains that cause IPD, the PCV may permit the use of antibiotics that have a stricter therapeutic use. These effects may be amplified by indirect protection. Additionally, the protection of PCV and reduction tendency of bacterial resistance – by reducing vaccine serotypes and consequently the carriage status load and IPD – may encompass the non-immunized population (herd effect).^29 , 30^

The study carried out in Peru did not report changes in antimicrobial resistance between the pre- and post-vaccine period. In the study performed in Venezuela, the authors did not find penicillin resistance after the implementation of PCV7. In Korea, a study was performed in 2010 after introducing PCV7 in 2003, in which nasopharyngeal aspirates were collected. The results of that study showed that the overall rate of non-susceptibility to penicillin increased from 83.5% to 95.4% (p=0.001). This finding may suggest that there was an increase of non-vaccine serotype PCV7 6A and 19A, which are resistant to antimicrobial agents.^31^ As a result of the effect of serotype replacement, it is expected an initial decrease in resistance to penicillin after PCV use. However, later, an increase in the non-resistant strain may occur as a consequence of non-vaccine serotype increase with this profile.^32^ Similar results were found in the United States, which had an increase in resistance to penicillin accompanied by a significant increase in non-vaccine serotype 19A after PCV7 implementation.^33^

Some limitations of our research should be considered. The limited number of studies that investigated the direct and indirect effects of the carriage status in children may underestimate our findings. In addition, the selected studies presented a small sample of nasopharyngeal. It was also not possible to perform a meta-analysis due to the great heterogeneity of the studies. The small number of studies selected could affect the understanding about the PCV impact on the LA&C countries, as well as demonstrate the need for future studies evaluating carrier status in the vaccinated and non-vaccinated populations. However, we understand that the selected studies could contribute to the scope of evidence of this review, by demonstrating the distribution of pneumococcal capsular types and describing the current scenario in the LA&C.

The PCV is one of the most expensive vaccines recommended by the Pan-American Health Organization (PAHO) and WHO. The replacement effect in PCV era was also observed. Thus, it is extremely important to know the current epidemiological profile of pneumococcus in the population after the introduction of PCV, so that policy makers can evaluate the real need to increase or not the valence of PCV in the national immunizations programs.

## CONCLUSION

The Latin American and Caribbean countries presented a decrease in vaccine serotypes in carriage status in children younger than 5 years. These countries also showed a replacement effect by non-vaccine serotype after pneumococcal conjugate vaccine administration. The distribution of the circulating serotypes is specific to each region and confers a distinct epidemiological profile for each country or region. Furthermore, the Latin American and Caribbean studies indicated a decrease in the resistance to antibiotics although others reports show an increase in the resistance in North America due to the replacement of non-vaccine serotype serotypes resistant to penicillin. Surveillance studies of pneumococcus carriage status should be continuous and aim to accompany the evolution of this microorganism in the post-pneumococcal conjugate vaccine scenarios. In this manner, such analyses may permit a more targeted strategy of control and prevention of invasive pneumococcal disease, particularly in the child population.
