# Supplementary material for: The direct and indirect effects of the pneumococcal conjugated vaccine on carriage rates in children aged younger than 5 years in Latin America and the Caribbean: a systematic review
Source: Einstein (Sao Paulo). 2019 Nov 8;18:eRW4890. doi: 10.31744/einstein_journal/2020RW4890 (PMC6896659; doi:10.31744/einstein_journal/2020RW4890)
Supplement: Translation

# Translation: Efeitos diretos e indiretos da vacina pneumocócica conjugada no estado de portador em crianças menores de 5 anos, na América Latina e no Caribe: uma revisão sistemática

**Authors:** Stefany Martins Silva, Izabella Caroline Gebrim Rodrigues, Rodrigo da Silva Santos, Yves Mauro Fernandes Ternes

**Affiliations:** 1 Universidade Federal de Goiás, Goiânia, GO, Brasil.

**Keywords:** Vacinas, Streptococcus pneumoniae, Portador sadio, Criança, América Latina

## Abstract

**Objetivo:**

Demonstrar o impacto das vacinas pneumocócicas conjugadas no estado de portador de *Streptococcus pneumoniae* em crianças menores de 5 anos na América Latina e no Caribe.

**Métodos:**

Foi realizada revisão sistemática da literatura sobre os efeitos diretos e indiretos da vacina pneumocócica no estado de portador em crianças menores de 5 anos, após a implantação da vacina nos calendários de imunização infantil. A partir de dados da PubMed^®^e da Biblioteca Virtual da Saúde, foram selecionados estudos de portador em crianças menores de 5 anos, com dados coletados após implementação da vacina de 2008 a 2018, na América Latina e no Caribe.

**Resultados:**

Dos 1.396 artigos identificados, 738 foram selecionados mediante leitura de títulos e resumos. Após a extração dos duplicados, 31 foram elegíveis para leitura do texto completo, restando 6 artigos para análise. Todos os estudos selecionados eram observacionais e indicavam diminuição do portador e tipos vacinais, e aumento da circulação de sorotipos não vacinais, como 6A, 19A, 35B, 21 e 38. Não foi observada alteração na resistência antimicrobiana após a introdução da vacina.

**Conclusão:**

Detectou-se redução no estado de portador, dos tipos vacinais e não vacinais. O monitoramento contínuo do efeito das vacinas pneumocócicas é fundamental, para demonstrar o impacto do estado de portador e, consequentemente, da doença pneumocócica invasiva, permitindo o melhor direcionamento nas ações em saúde para os países que incluíram a vacina no calendário de imunização. Nosso protocolo de estudo foi registrado no PROSPERO (www.crd.york.ac.uk/prospero) sob o número CRD42018096719.

## INTRODUÇÃO

A Organização Mundial da Saúde (OMS) caracteriza as enfermidades respiratórias, notadamente as pneumocócicas, como as principais causas de morbidade e mortalidade na infância.^1 , 2^ A vacina pneumocócica conjugada (VPC) é um avanço notável para a saúde pública, sendo o principal método de prevenção contra doenças pneumocócicas em populações vulneráveis, como crianças abaixo de 5 anos de idade.^2^

A OMS sugere que os países em desenvolvimento sejam priorizados em um esforço para reduzir a taxa de mortalidade de crianças menores de 5 anos, pois essa população é frequentemente imunocomprometida e desnutrida. Além disso, esses indivíduos são de família de baixa renda, têm várias comorbidades e, geralmente, vivem em condições precárias. Esses fatores indicam que o acréscimo da VPC aos programas de imunização infantil dos países em desenvolvimento beneficiaria notavelmente essa população.^3^ Em 2010, o Brasil foi um dos primeiros países latino-americanos a incluir uma VPC, a vacina pneumocócica 10-valente (VPC10), em seu programa nacional de vacinação. A VPC10 é composta pelos dez principais sorotipos de *Streptococcus pneumoniae* (pneumococo) mais frequentemente associados a doenças pneumocócicas no país.^4^ Outros países latino-americanos, como o Chile, estabeleceram o uso da vacina pneumocócica 13-valente (VPC13) ao seu programa de vacinação, em 2010.^3^

*S. pneumoniae* é importante patógeno respiratório capaz de colonizar a mucosa do trato respiratório e a região nasofaríngea. Esse estado de portador pode durar de semanas a meses. O patógeno apresenta uma cápsula polissacarídica com mais de 90 sorotipos capsulares^5 , 6^ e pode ser transmitido pelo contato direto com secreções ou pela dispersão de aerossóis efetuada pelos indivíduos afetados.^7^

Após a implementação da VPC nos programas nacionais de vacinação, espera-se redução no estado de portador do pneumococo, o que pode reduzir a carga de infecção por pneumococo entre as crianças. Consequentemente, o impacto no estado de portador pode resultar em cenário de substituição de sorotipo.^8^ Reunindo essas hipóteses, o objetivo deste estudo foi investigar os efeitos diretos e indiretos do estado de portador do pneumococo em crianças abaixo de 5 anos de idade. A determinação da colonização da nasofaríngea pelo pneumococo nessa população pode gerar insights para vigilância e políticas estratégicas nos países que implementaram o VPC em seus programas nacionais de vacinação.

## OBJETIVO

Demonstrar o impacto da vacina pneumocócica conjugada no estado de portador de *Streptococcus pneumoniae* em crianças abaixo de 5 anos de idade na América Latina e no Caribe.

## MÉTODOS

Foi realizada uma revisão sistemática da literatura com estudos realizados na América Latina e Caribe (ALC), publicados entre janeiro de 2008 e fevereiro de 2018. Selecionamos publicações científicas relacionadas ao impacto (efeitos diretos e indiretos) da vacina pneumocócica no estado de portador de pneumococo em crianças após a implementação de VPC nos programas de imunização infantil. Os grupos etários alvos da VPC foram avaliadas efeito direto; os grupos etários que não foram alvos da VPC foram considerados efeito indireto da vacina.

As buscas foram realizadas nos bancos de dados *National Center for Biotechnology Information, National Library of Medicine* (NCBI/PubMed^®^) e Biblioteca Virtual em Saúde (BVS). Não empregamos restrições de idioma para os estudos publicados. Não foram avaliadas publicações que não estivessem totalmente disponíveis nas plataformas.

### Critérios de inclusão e exclusão

Investigamos estudos realizados em países da ALC e publicados entre 2008 e 2018, desde que a coleta de dados fosse realizada após a implementação da VPC. Para elegibilidade de nossa revisão de literatura, avaliamos exclusivamente estudos realizados com crianças abaixo de 5 anos de idade e que contivessem resultados de estado de portador. Para tanto, consideramos apenas estudos observacionais.

Excluímos de nossas análises artigos de revisão, estudos randomizados e experimentais, estudos realizados em países não localizados na ALC, publicações que incluíssem crianças acima de 5 anos de idade, estudos publicados antes de 2008 e que contivessem resultados para doenças invasivas.

### Processo de seleção

Para selecionar os estudos das bases de dados das publicações, foram utilizadas as seguintes palavras-chave: (pneumococcal vaccine AND children AND carriage) [vacina pneumocócica AND crianças AND portador] OR (pneumococcal vaccine AND children AND colonizing) [vacina pneumocócica AND crianças AND colonizar] OR ( *Streptococcus pneumoniae* vaccine AND children AND carriage) [ *Streptococcus pneumoniae* AND crianças AND portador] OR ( *Streptococcus pneumoniae* vaccine AND children AND colonizing) [ *Streptococcus pneumoniae* AND crianças AND colonizar]. Os estudos selecionados foram analisados por dois revisores independentes. Preparamos dois formulários que foram usados para estratificar os manuscritos selecionados com base nos relatórios da Professional Society for Health Economics and Outcomes Research (ISPOR). No Formulário de Extração de Dados Nível 1, os estudos foram selecionados com base nos títulos e resumos, usando os critérios de exclusão mencionados. Os estudos selecionados foram, então, submetidos à análise por meio do Formulário de Extração de Dados Nível 2, baseada na triagem de textos na íntegra. Durante a análise de elegibilidade, um terceiro revisor analisou os estudos em que houve discordância entre os dois primeiros revisores. Os formulários 1 e 2 estão disponíveis no material suplementar.

## RESULTADOS

A figura 1 mostra o fluxo para identificação e seleção de estudos. Identificamos 1.396 estudos e excluímos 658 duplicatas. Após a triagem com o Formulário de Extração de Dados Nível 1, de 738 estudos investigados, 31 atenderam a todos os critérios exigidos e foram, então, submetidos ao Formulário de Extração de Dados Nível 2. Dos 31 relatos, 25 foram caracterizados como potencialmente elegíveis, pouco claros ou excluídos, resultando em 6 estudos para análise. Dos seis estudos selecionados, três foram realizados no Brasil, um na Colômbia, um na Venezuela e um no Peru. A maioria dos estudos (quatro estudos) comparou os períodos pré e pós-vacinação com VPC diferentes. Dessa forma, o efeito individual de cada país/VPC deve ser levado em conta como um meio de sugerir o aumento da valência da vacina, sem comparar o efeito entre os países.

**Figura 1:** Fluxograma de seleção dos estudos NCBI: *National Center for Biotechnology Information* ; BVS: Biblioteca Virtual em Saúde.

As características de cada estudo são mostradas na tabela 1 . Os estudos brasileiros investigaram os efeitos após a introdução da VPC10 no Programa Nacional de Imunização Infantil, em 2010.^9^ O estudo da Venezuela foi realizado com amostras de nasofaríngea de 84 crianças com menos de 5 anos de idade, vacinadas com o VPC7.^10^ A vacina conjugada contra o pneumococo 7, distribuída em todo o território latino-americano, ainda não estava presente no programa de imunização da Venezuela. Entretanto, foram investigados seus benefícios, efeitos e sorotipos encontrados em circulação. Em 2014, a VPC13 foi implementada no programa de imunização e oferecida à população infantil da Venezuela.^11 , 12^ Os estudos da Colômbia e do Peru caracterizaram os efeitos da VPC7 após a introdução dessa vacina no programa de imunização de ambos os países, em 2009.^13 , 14^

**Tabela 1:** Efeitos diretos e indiretos dos estudos selecionados

Referência	País (cidade)	População (n)^pós-vacina^	Vacina (anos de investigação)	Efeito	Resistência a antibióticos
Bello González et al.^(10)^	Venezuela (Bolívar)	<5 anos (n=84)	VPC7^*^	Elevada prevalência do sorotipo não vacinal 33F (21,5%) nas taxas de portador	Não foi detectada resistência à penicilina
			Pós-vacina (2008)		
Parra et al^.(13)^	Colômbia (Bogotá)	12-8 meses (n=197)	VPC7	Redução do sorotipo vacinal^†^VPC& (23,6% em crianças não imunizadas para 7,6% em crianças imunizadas)	Verificada resistência para doenças invasivas
			Pré- e pós-vacina (2005-2009/2011)		

				Aumento do sorotipo não vacinal^‡^(24% em crianças não imunizadas para 34% em crianças imunizadas)	
Hanke et al.^(14)^	Peru (São Marcos)	Até 2 anos (n=125)	VPC7	Redução do sorotipo vacinal^†^VPC7 (48% → 28,8%) em crianças imunizadas	A resistência a antibióticos não alterou após a implementação da vacina
			Pré- e pós-vacina (2009/ 2011)		
				Aumento do sorotipo não vacinal^‡^(52% → 71,2%) em crianças imunizadas	
Andrade et al.^(16)^	Brasil (Goiânia)	7-11 meses/15-18 meses (n=1.287)	VPC10	Redução do estado de portador	Não foi verificada resistência a antibióticos
			Pós-vacina (2010)	Redução do sorotipo vacinal^†^de VPC10	
Brandileone et al.^(17)^	Brasil (São Paulo)	12-23 meses (n=400)	VPC10	Redução no estado de portador (>90%)	Não foi verificada resistência a antibióticos
			Pré- e pós-vacina (2010/ 2013)	Redução do sorotipo vacinal^†^de VPC10	
Andrade et al.^(18)^	Brasil (Salvador)	6-23 meses (n=53)	VPC10	Nenhuma mudança foi observada no estado de portador	Não foi verificada resistência a antibióticos
			Pré- e pós-vacina (2009/ 2013)		

^*^Após o uso da VPC7 no território, desde que a VPC13 foi implementada em 2014 no programa de imunização;^†^sorotipo vacinal;^‡^sorotipo não vacinal.

VPC: vacina pneumocócica conjugada.

As crianças que viviam em regiões isoladas da Bolívia e da Venezuela apresentaram maior prevalência de *S. pneumoniae* entre 1 e 2 anos de idade (82%). O sorotipo 6B foi mais frequente (49%), seguido pelos sorotipos 33F (21,5%), 19F (3,1%) e 23F (1,3%). Os sorotipos 6B e 23F estavam presentes na vacina VPC7, denominados sorotipos vacinais, totalizando 51% dos sorotipos encontrados na nasofaríngea dessa população. Entretanto, o sorotipo não vacinal 33F apresentou alta incidência e foi identificado na maioria das comunidades investigadas. Essa observação é altamente relevante, uma vez que os índices do sorotipo 33F estão aumentando no mundo.^10 , 15^

As associações entre a distribuição dos sorotipos de pneumococo e o estado de portador são mostradas na figura 2 . Todo o conjunto de estudos selecionados mostrou diminuição significativa na prevalência de sorotipos vacinais no portador após a implementação da VPC, independentemente da vacina (VPC7, VPC10 ou VPC13). Além disso, o efeito de substituição foi evidente, uma vez que todos os estudos encontraram aumento na prevalência dos sorotipos não vacinais.

**Figura 2:** Perfil da prevalência de sorotipos vacinais e não vacinais de pneumococos TV: tipo vacinal; TNV: tipo não vacinal.

Investigando o impacto da resistência microbiana no estudo da Venezuela, nenhuma das cepas era resistente à penicilina. Além disso, 13,7% dos pacientes eram resistentes aos macrolídeos, 3,4% à tetraciclina (TET) e 3,4% ao sulfametoxazol-trimetoprima (STX-TMP), não sendo encontrada multirresistência nos isolados avaliados.^10^

## DISCUSSÃO

Até onde sabemos, esta é a primeira revisão sistemática do impacto da VPC no estado de portador de pneumococos em crianças abaixo de 5 anos de idade, na América Latina e Caribe. Em nosso estudo, demonstramos que a VPC levou a uma redução do estado de portador, o que também promoveu menor circulação do pneumococo sorotipo vacinal na população infantil. A redução do estado de portador influencia diretamente no desenvolvimento e na mortalidade como resultado da doença pneumocócica invasiva (DPI).^19 - 21^

Em geral, os estudos evidenciaram redução no sorotipo vacinal e aumento no sorotipo não vacinal.^13 , 14 , 16 , 17^ O sorotipo não vacinal 33F foi encontrado exclusivamente na Venezuela, com prevalência significativa.^10^ Além disso, o Brasil apresentou exclusivamente o sorotipo não vacinal VPC10 19A, que aumentou no período pós-vacinação, assim como os índices de sorotipo não vacinal 23A na Colômbia.^13 , 16^ De modo semelhante, nos Estados Unidos houve substituição do sorotipo vacinal pelo sorotipo não vacinal, com aumento nos sorotipos 6C, 15A, 15B, 15C, 23A e 35B.^22 , 23^ Na Europa, estudos da Noruega e Alemanha mostraram que os sorotipos não vacinais 15A e 23B estavam aumentando em frequência.^24 , 25^ Na França, foram detectados os sorotipos não vacinais 12F, 15A, 24F e 35B.^26^ Na Ásia, o Japão também teve um aumento dos sorotipos não vacinais, como 19A, 15A, 15B, 15C, 22F e 24F, após a implementação da VPC7, com aumento do subtipo sorotipo não vacinal de 8,0% para 48,1%.^27^

Assim, apesar do sucesso da VPC em reduzir a aquisição de pneumococos que colonizam a nasofaríngea e, consequentemente, promovem aumento na carga de DPI, o perfil etiológico da VPC pode ser alterado devido ao aumento do estado de portador por sorotipos ausentes nas vacinas. Essa alteração de perfil das VPCs é chamada de substituição.

O uso de vacinas na prevenção da DPI promoveu diminuição na colonização e na circulação de agentes bacterianos, resultando na redução do uso de medicamentos antimicrobianos. Dessa forma, é possível diminuir a prevalência de bactérias resistentes ou multirresistentes.^28^ Ao reduzir o número de cepas resistentes que causam DPI, a VPC pode permitir a utilização de antibióticos que tenham uso terapêutico mais estrito. Esses efeitos podem ser amplificados por proteção indireta. Além disso, a proteção da VPC e a tendência de redução da resistência bacteriana — pela redução dos sorotipos vacinais e, consequentemente, da carga de estado de portador e da DPI — podem abranger a população não imunizada (efeito rebanho).^29 , 30^

O estudo realizado no Peru não relatou mudanças na resistência antimicrobiana entre o período pré e pós-vacinação. No estudo realizado na Venezuela, os autores não encontraram resistência à penicilina após a implementação da VPC7. Na Coreia, um estudo foi realizado em 2010 após a introdução da VPC7 em 2003, no qual foram coletados aspirados de nasofaríngea. Os resultados desse estudo mostraram que o índice geral de não suscetibilidade à penicilina aumentou de 83,5% para 95,4% (p=0,001). Esse achado pode sugerir que houve aumento do sorotipo não vacinal VPC7 6A e do 19A, que são resistentes aos agentes antimicrobianos.^31^ Como resultado do efeito da substituição do sorotipo, é esperada a diminuição inicial na resistência à penicilina após o uso da VPC. No entanto, mais tarde, pode ocorrer aumento na cepa não resistente como consequência do aumento do sorotipo não vacinal com esse perfil.^32^ Resultados semelhantes foram encontrados nos Estados Unidos, onde houve aumento na resistência à penicilina, acompanhada pelo aumento significativo no sorotipo não vacinal 19A após a implementação da VPC7.^33^

Algumas limitações de nossa pesquisa devem ser levadas em consideração. O número limitado de estudos que investigaram os efeitos diretos e indiretos do estado de portador em crianças pode subestimar nossos achados. Além disso, os estudos selecionados apresentaram pequena amostra de nasofaríngea. Não foi possível realizar metanálise devido à grande heterogeneidade dos estudos. O pequeno número de estudos selecionados poderia afetar o entendimento sobre o impacto da VPC nos países da América Latina e Caribe, bem como demonstrar a necessidade de estudos futuros, que avaliem o estado de portador nas populações vacinada e não vacinada. No entanto, entendemos que os estudos selecionados poderiam contribuir para o escopo de evidência desta revisão, demonstrando a distribuição dos tipos capsulares do pneumococo e descrevendo o cenário atual na ALC.

A VPC é uma das vacinas mais caras recomendadas pela Organização Pan-Americana de Saúde (OPAS) e pela OMS. Observou-se também o efeito de substituição na era da VPC. Assim, é de extrema importância conhecer o perfil epidemiológico atual do pneumococo na população após a introdução da VPC, para que os formuladores de políticas possam avaliar a real necessidade de aumentar ou não a valência da VPC para os programas nacionais de imunização.

## CONCLUSÃO

Os países da América Latina e Caribe apresentaram diminuição dos sorotipos vacinais no estado de portador em crianças abaixo de 5 anos de idade. Esses países também mostraram efeito de substituição pelo sorotipo não vacinal após a administração da vacina pneumocócica conjugada. A distribuição dos sorotipos circulantes é específica para cada região e confere perfil epidemiológico distinto para cada país ou região. Ademais, os estudos da América Latina e do Caribe indicaram diminuição na resistência a antibióticos, embora outros relatos mostrem aumento na resistência na América do Norte, devido à substituição por sorotipos não vacinais resistentes à penicilina. Estudos de vigilância do estado de portador de pneumococos devem ser contínuos e visam acompanhar a evolução desse microrganismo nos cenários pós-vacinas pneumocócicas conjugadas. Dessa forma, tais análises podem permitir estratégia mais direcionada de controle e prevenção da doença pneumocócica invasiva, particularmente na população infantil.
